# Non-linear susceptibility to interferences in declarative memory formation

**DOI:** 10.1371/journal.pone.0270678

**Published:** 2022-06-29

**Authors:** Malen D. Moyano, Giulia Carbonari, Matías Bonilla, María E. Pedreira, Luis I. Brusco, Laura Kaczer, Cecilia Forcato

**Affiliations:** 1 Laboratorio de Sueño y Memoria, Departamento de Ciencias de la Vida, Instituto Tecnológico (ITBA), Buenos Aires, Argentina; 2 Consejo Nacional de Investigaciones Científicas y Tecnológicas (CONICET), Buenos Aires, Argentina; 3 IFIBYNE-CONICET, Facultad de Ciencias Exactas y Naturales, Universidad de Buenos Aires, Buenos Aires, Argentina; 4 Centro de Neuropsiquiatría y Neurología de la Conducta-CENECON, Facultad de Ciencias Médicas, Universidad de Buenos Aires, Buenos Aires, Argentina; Nathan S Kline Institute, UNITED STATES

## Abstract

After encoding, memories go through a labile state followed by a stabilization process known as consolidation. Once consolidated they can enter a new labile state after the presentation of a reminder of the original memory, followed by a period of re-stabilization (reconsolidation). During these periods of lability the memory traces can be modified. Currently, some studies show a rapid stabilization after 30 min, while others show that stabilization occurs after longer periods (e.g. > 6 h). Here we investigate the effect of an interference treatment on declarative memory consolidation, comparing distinct time intervals after acquisition. On day 1, participants learned a list of non- syllable pairs (List 1). 5 min, 30 min, 3 h or 8 h later, they received an interference list (List 2) that acted as an amnesic agent. On day 2 (48 h after training) participants had to recall List 1 first, followed by List 2. We found that the List 1 memory was susceptible to interference when List 2 was administered 5 min or 3 h after learning but not when it was administered 30 min or 8 h after. We propose the possibility that this rapid memory protection could be induced by a fast and transient neocortical integration. Our results open a discussion about the contribution of molecular and systemic aspects to memory consolidation.

## Introduction

Memory consolidation encompasses different processes at multiple levels of organization in the brain. From the molecular to the behavioral level and over a temporal spectrum ranging from seconds to months and years, this process transforms, stabilizes and updates memory traces according to contextual demands [1]. Consolidation involves modifications of the synapses concerning the engram (synaptic consolidation) as well as a redistribution of the information to long-term storage areas (system consolidation) [1]. However, once consolidated, memories are not fixed. After the presentation of a cue associated with the original information (reminder), stored memories can return to a labile state followed by a process of re-stabilization (reconsolidation), dependent on protein synthesis and gene expression [2]. Even though reconsolidation is not a recapitulation of the consolidation process, they share common molecular mechanisms [3] as well as similar time windows in which memory is sensitive to interferences (after learning or after the presentation of the reminder) [2,4–8]. Furthermore, during these labile periods, memories can not only be impaired, but also be strengthened or updated in content [9–11].

There is a consensus in the neurobiology of memory field that shortly after acquisition/reactivation (e.g. 5 min or immediately after) interfering tasks can impair memory consolidation/reconsolidation, but that after longer intervals (e.g. > 6 h) memories are protected against interference [2,4–6]. These results support the hypothesis that memory consolidation/reconsolidation are linear processes accomplished around 6 h after acquisition/reactivation. After this period of time, memory would no longer be modified without external reactivation and thus, could be protected against interferences [2,4]. In the same line, other findings showed similar results, however, with a shorter time window of consolidation/reconsolidation, around 30–40 min after acquisition/reactivation [7,8]. However, it is important to highlight that several of these studies do not test longer intervals. Thus, it is not possible to discard that consolidation/reconsolidation could have a second time window of susceptibility to interference at a later time. Taking all this in mind, a question that emerges is whether the resistance to interference is a gradually emergent property after acquisition/reactivation, or if there is more than one time window of susceptibility instead. In line with this, Dudai and Eisenberg [12] and Alberini [3] proposed the existence of multiple waves of consolidation processes that are initiated after acquisition and can be modulated by either implicit (internal reactivations) or explicit reactivations (cued-recall) instead of only one time window where the memory is labile and afterward stabilized. Thus, in the present study, we aim to dissect the temporal dynamics of the consolidation process, using the susceptibility to interference as a tool to reveal memory stabilization, given that one of the hallmarks of the memory stabilization process is the vulnerability of memory to amnesic agents, including retroactively interfering stimuli [13–15]. From a neurobiological perspective, if an interference task shares the same memory system than the target memory, it implies a competition for shared resources between the original and new memory [13]. So, manipulating the interval between the acquisition of the target memory and the interference task allows one to obtain a profile of the susceptibility to interferences that could hint towards the neurobiological basis of memory consolidation [7].

Here, we use a nonsense syllable paradigm that has been well-characterized by our research group, especially regarding the reminder structure required to trigger memory reconsolidation, memory improvement and updating [4,16–18]. We tested whether protection against interference of declarative memories is a non-linear process that emerges after acquisition. For that, we performed a two-day experiment with six groups. Participants learned a list of five pairs of non-syllable pairs [14] on day 1 (List 1). 5 min after learning, 30 min, 3 h or 8 h later they received an interference task (List 2) that acted as an amnesic agent. They were finally tested on both lists on day 2 (48 h after training). As an indirect measurement to examine memory interference we use the Retrieval-Induced Forgetting effect (RIF, [19,20]). In this sense, we have previously demonstrated that an impaired stabilization of the target memory was typically indicated by the absence of a RIF effect on List 2 recall [4]. Thus, the RIF effect allows for an assessment of the integrity of the memory trace, i.e. whether a stored memory is intact or impaired, beyond simple retrieval performance. According to the RIF effect, retrieval of one memory (e.g. List 1) can temporarily block the retrieval of a related memory (e.g. List 2, [4]). This RIF effect is only observed when the memory trace that is recalled in the first place is intact, whereas the RIF effect is not observed when the first recalled memory trace is impaired. Thus, here we revealed memory consolidation impairment by the absence of RIF effect on List 2 memory recall.

## Materials and methods

### Participants

215 volunteers were enrolled in the study (163 women, 52 men). The participants were undergraduate and graduate students with ages ranging from 18 to 36. They were recruited via mail and social media pages from our laboratory (Twitter, Facebook and Instagram). To be able to participate, they first had an interview with the experimenter who explained the procedures and next they had to tick a box in an online consent form approved by the “Comité de Ética en Investigación Biomédica del Instituto Alberto C. Taquini”, in accordance to the principles expressed in the Declaration of Helsinki. Among the participants that concluded the experiment, three gift cards from a bookstore were raffled. None of the participants reported ongoing medication, health problems, medical interventions, or history of psychiatric, neurological, or sleep disorders.

Subjects that reached at least 55% of correct responses during the last four training trials (11/20 correct responses) were included in the analysis [21]. 77 subjects were excluded from the analysis because they did not reach the learning criterion (43), did not followed/understand the instructions of the task (16), wrote the syllables in a piece of paper during the training session or the retention interval (10), used psychopharmaceuticals that they had not reported it the initial interview (4), slept a nap during retention interval (1), did not accomplished the schedule for carry out the experiment (2) or technical problems (1). The final sample included 138 participants (102 women), with ages ranging from 18 to 36 (25.74 ± 0.45 years).

### Experimental groups

**Fig 1:**
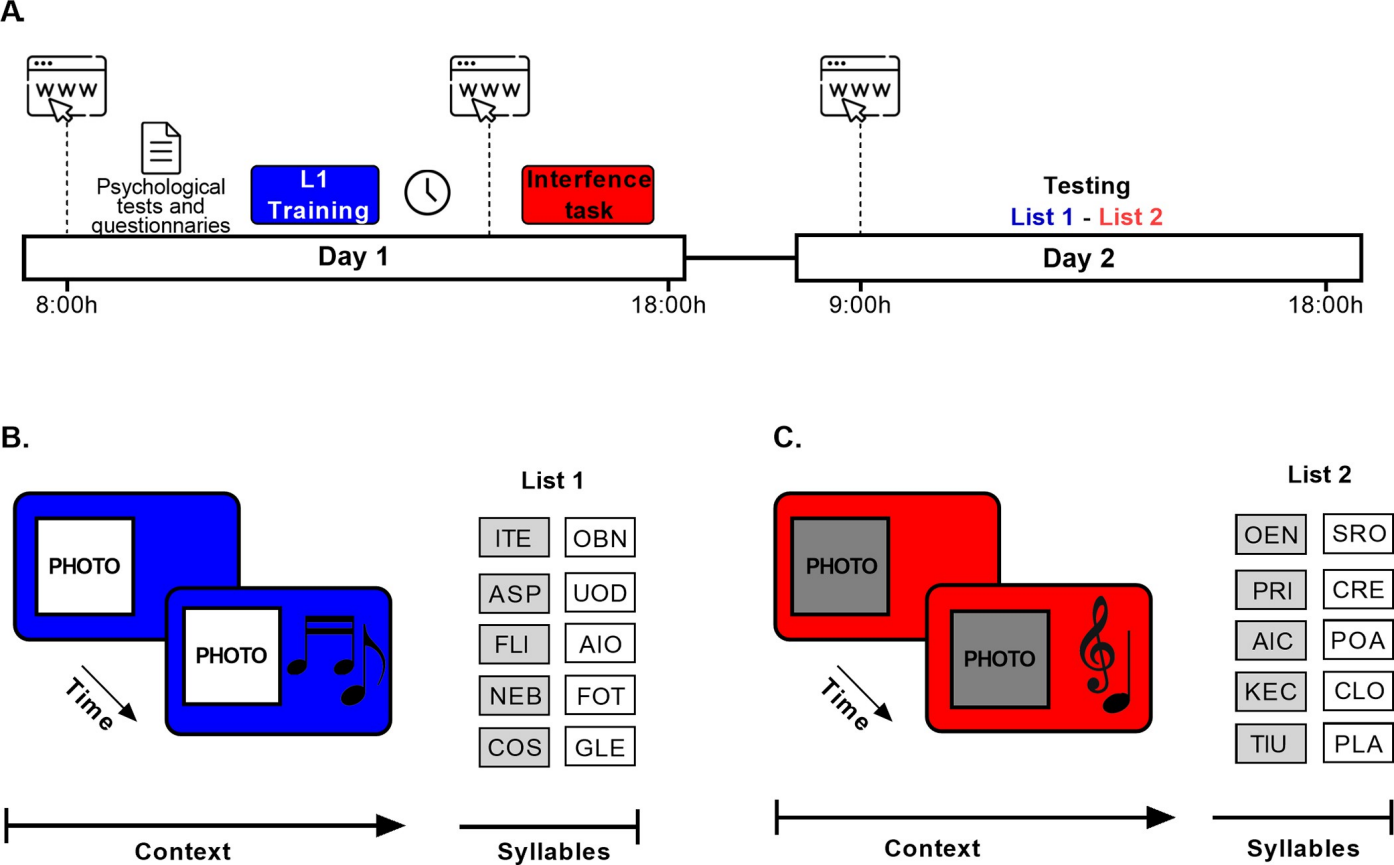
Experimental design and memory task. (A) Protocol. On day 1, the participants started the experiment between 9:00 h and 18:00 h. First, they gave their informed consent, filled out a personal data questionnaire and completed the psychological tests and questionnaires (Beck Depression Inventory II, State- Trait Anxiety Inventory, Pittsburgh Sleep Quality Index and Morningness-Eveningness Questionnaire). Next, all the experimental groups received the List 1 training. The groups differed in the moment they received the interference task (5 min, 30 min, 3 h or 8 h after List 1 training). On day 2, at 48 h on day 1, all groups received an email at 9:00 h with the link to re-enter the platform and performed the last session of the experiment. They could enter the link at any time between 9:00 h and 18:00 h. The experimental groups were tested for retrieval on both lists: First for List 1 and then for List 2, while the control groups were only tested on the single list. (B). List 1 Training. The training session consisted of 10 trials. Each trial started with the presentation of the blue background and image of an Italian coast for 4 sec, followed by the same stimuli accompanied by the tarantella music for another 4 sec. After that, the five pairs of cue-response syllables (List 1) were presented successively and in random order. (C). Interference task. The training of the interfering task was the same as in B but with a different context (red background color, the image of a forest and classical music) and different five pairs cue-response syllables (List 2).

Participants were randomly assigned to one of six groups. All groups performed the List 1 training on day 1 and were tested on day 2 (Fig 1A). The groups differed in the moment they received the interference task (List 2) on day 1. The “G-5min” group (n = 23) received the interference task 5 min after the List 1 training. The “G-30 min” group (n = 23) received the interference task 30 min after the List 1 training. The “G-3h” group (n = 23) learned the List 1, and received the interference task 3 h later. The “G-8h” group (n = 23) received the interference task 8 h after the List 1 training. Two control groups were assessed: The control group for the List 1 (“CTL-L1”, n = 23) received the List 1 training on day 1 but did not receive the interference task and were tested on day 3. Participants in the control group for the List 2 (“CTL-L2”, n = 23) only learned the interference task on day 1 and were tested on day 3 for that list.

### Experimental design

The experiment was conducted online due to the Covid-19 pandemic. We used Gorilla Experiment Builder (www.gorilla.sc) to create and host our experiment [22]. The participants gave their informed consent, filled out a personal data questionnaire and completed a series of psychological tests and questionnaires (Beck Depression Inventory II, State- Trait Anxiety Inventory, Pittsburgh Sleep Quality Index and Morningness-Eveningness Questionnaire). After that, they received the instructions for the memory task and completed a demo test. Then the procedure changed for each group (Fig 1A). The “G-5min” group learned the first list of syllable-pairs (List 1) and 5 min after learned the interference task (List 2). The “G-30min” group learned the List 1 and after 30 minutes they received an email to re-enter the platform and learned the interference task. The “G-3h” group followed the same procedure as the “G-30min” group, except that they had a 3 h period between the List 1 and the List 2 training. The “G-8h” group learned the List 1 and they had an 8 h period between the List 1 and List 2 training. Both the "G-3h" group and the "G-8h" group continued with their normal activities during the 3 h or 8 h, respectively. Moreover, they were instructed not to sleep during this period. The procedures for the control groups were similar to the experimental groups but they only learned one list of syllables. The “CTL-L1” group learned the List 1 and the “CTL-L2” group learned the List 2. On day 2 (48 h later) all groups received an email with the link to re-enter the platform and performed the last session of the experiment. The experimental groups were tested on both lists: first for List 1 and then for List 2, while the control groups were only tested on the single list they had learned.

### The task

The task consisted of memorizing five pairs of nonsense syllables, associated with a context formed by a background color on the computer screen, an image and music, presented through headphones [23]. The syllables were formed by three letters (Fig 1B and 1C). We have previously shown that including a context associated with the list of syllables improved memory retention experiment [4].

#### L1 training

List 1 was constituted by five pairs of nonsense cue-response syllables: **ITE**-OBN, **ASP**-UOD, **FLI**-AIO, **NEB**-FOT, **COS**-GLE (bold type: cue-syllable; regular type: response-syllable, Fig 1B). The training session of List 1 consisted of 10 trials, associated with a context (Fig 1B). The first trial started with the presentation of the context: first, the image of an Italian coast with a blue background appeared on the screen for 4 sec. Then, a tarantella melody played along with the image and background color for another 4 sec. After that, the context continued while the syllables were presented. First, one cue-syllable appeared at the left top side of the monitor’s screen and an empty response box was displayed on the right top. Next, the corresponding response-syllable appeared in the response box and stayed there for 4 sec. Immediately thereafter, the syllable pair disappeared and another cue-syllable was shown one line below and the process was repeated until the list was complete. The presentation order was randomized. In the successive nine trials subjects were required to write down the corresponding response- syllable for each cue-syllable presented. The cue-syllable appeared at the left top side of the monitor’s screen and an empty response box on the right top. Subjects were given 5 sec to write the corresponding response-syllable. If no syllable was written down, the correct one was shown for 4 sec; if an incorrect syllable was written, it was replaced by the correct one and it was shown for 4 sec; and if the correct response was given, it stayed for 4 sec. Immediately thereafter, the syllable pair disappeared and another cue-syllable was shown one line below and the process was repeated until the list was complete. After the whole list was presented, a black background was shown for 3 sec and the procedure was repeated until the entire list of syllable-pairs was completed nine times. The List 1 training session took about 10 min.

#### List 2 training (Interference task)

The interference task consisted of learning another list of syllable pairs, List 2, formed by five different pairs of nonsense cue-response syllables: **OEN**-SRO, **DRI**-CRE, **AIC**-POA, **TIU**-PLA, **KEC**-CLO (Fig 1C). Learning of the interference task was similar to the List 1 training, but with a different context (red background color, the image of a forest and classical music). Like the List 1 training, it was formed by 10 trials. In the first trial, participants observed how the five syllable pairs were completed once and in the successive nine trials they had to write down the corresponding response-syllables. Feedback procedures were the same as for List 1 training. The interference task took about 10 min.

#### Testing

On day 2, List 1 and List 2 memory were tested. List 1 memory was always tested first. For both lists, the testing session was formed by four trials each, similar to the training session, but subjects were required to write down the corresponding response-syllable within 5 sec. Cue-syllables were presented randomly. Testing took about 8 min (4min per List). Written responses were registered at the finish of List 1 training, List 2 training, and testing

#### Demo

Before the List 1 training, participants were presented with a demo program to receive all the instructions and to make sure that all participants understood the task. The demo program consisted of two trials, similar in structure as the training session but with another context and two different pairs of nonsense syllables.

### Tests and questionnaires

The study was carried out during quarantine by COVID-19 in Argentina. Preventive isolation increased levels of stress, anxiety and depression significantly in the population [24]. Considering that an increase in the values of negative mental symptoms such as anxiety and depression, can modulate the encoding, consolidation, and retrieval of memories [25–29], we evaluated psychological variables using Beck Depression Inventory II (BDI-II [30]) and State-Trait Anxiety Inventory (STAI [31]). Thus, we corroborate that all groups had the same level of anxiety and depression. Also, we evaluated quality sleep using Pittsburgh sleep quality index (PSQI [32]) and chronotype using Morningness-Eveningness Questionnaire (MEQ, [33]).

### Data analysis and statistics

Statistical analyses were performed using SPSS version 21 (IBM Corporation). We counted the number of correct responses reached in the last training trial of each list as a measure of the level of training, and also the number of correct responses at the first testing trial of each list as a measure of memory retention. We further calculated the memory change as the number of correct responses at testing minus the number of correct responses at training. Thus, positive values mean memory gain and negative values, memory loss. We also counted the type of errors at the last training trial and the first testing trial of each list and calculated the memory change for each type of error. We first analyzed the number of correct responses at List 2 training and testing with a repeated measures ANOVA with “group” as an inter-subject factor with 5 levels (“CTL2”, “G-5min”, “G-30 min”, “G-3 h” and “G-8 h”) and “session” as a repeated measure with 2 levels (last training trial and first testing trial). After significant interaction, we performed simple effect analysis followed by pairwise comparisons corrected by Bonferroni’s adjustment for multiple comparisons. The same analysis was performed for List 1.

The memory change for each List was analyzed with one-way ANOVA with “group” as a between subjects factor followed by Bonferroni Post-hoc comparisons. Comparisons in each analysis were made against the control group of each list. For all analyses, we set an alpha value of 0.05.

We further analyzed the State Anxiety, Trait Anxiety, BDI-II, PSQI and MEQ in all conditions with one-way ANOVA with “group” as a between subjects factor.

In order to analyze the dynamics of the RIF effect on List 2 memory change, based on the data obtained for all time points (5 min, 30 min, 3 h, 8 h), we fitted them to a cubic function using a cubic spline interpolation in Python. Also, to evaluate the dynamics of the interference task on the List 1 memory change, all time points were fitted to another cubic spline function.

We reported partial eta square (η^2^_p_) and Cohen’s d as effect size estimates.

#### Type of errors

We classified the errors into three categories: “void”, when no response was written down; “confusion”, when the written response-syllable was not included in any of the lists and; “intralist”, when a wrong response-syllable from the same list was written down experiment [16].

The memory change of the different types of errors was analyzed with one-way ANOVAs followed by Bonferroni Post-hoc comparisons. For the non-parametric variables, we used the Kruskal-Wallis Test followed by Games-Howell Post-hoc comparisons.

### Method to test List 1 memory trace impairment

**Fig 2:**
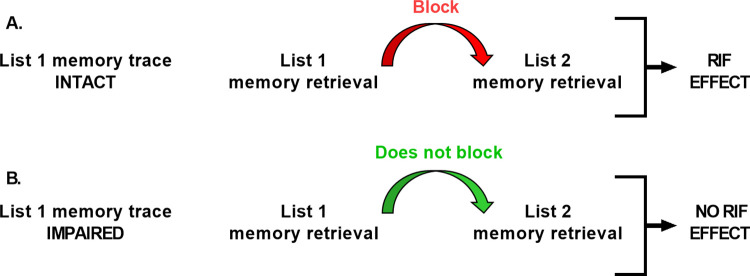
Method to test List 1 memory trace impairment. According to the Retrieval- Induced Forgetting effect (RIF), retrieval of one memory can temporarily block subsequent retrieval of other related memories. (A) RIF effect is only observed when the memory trace that is recalled first is intact (B) RIF effect is not observed when the first recalled memory trace is impaired. Anderson et al., 1994; MacLeod & Macrae, 2001, Forcato et al., 2007.

Memories are integrated into complex associative networks, and accordingly, the activation of one memory can activate different traces simultaneously (e.g. List 1 and List 2). So, we analyzed the Retrieval- Induced Forgetting (RIF) effect [19,20] in two ways: analyzing the number of correct responses at List 2 testing and also analyzing the List 2 memory change. Both ways indicate to what extent the prior retrieval of the target memory (List 1) blocks the subsequent retrieval of related List 2 memory. The presence of the RIF effect, i.e. fewer List 2 correct answers than the control group or more memory change (memory decay between List 2 training and List 2 testing than the control group) indicates a stable List 1 memory trace (Fig 2A). On the other hand, the absence of the RIF effect, i.e. unaffected List 2 retrieval (no difference in the correct answers compared to the control group or similar memory change than the control group) indicates List 1 memory trace impairment (Fig 2B [4,16]).

In addition, memory performance at retrieval may be influenced by simultaneous retrieval interference. If memories of both List 1 and List 2 are recalled during the same session at retrieval, they may interfere with each other leading to acute performance decrements, which are, however, independent of the integrity of the underlying memory trace [4,16,23].

## Results

### List 2 performance

**Fig 3:**
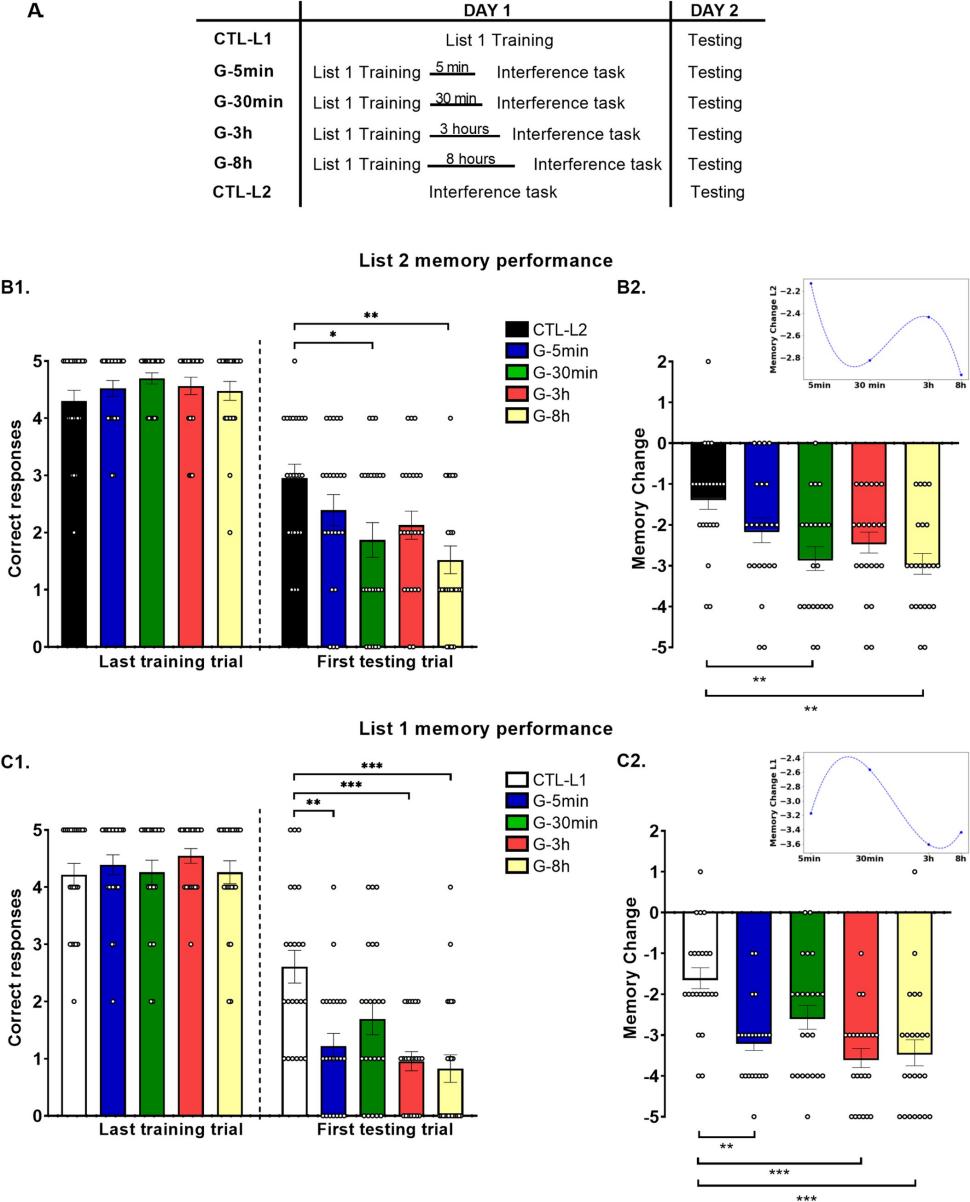
List 2 and List 1 memory performance. (A) Experimental groups. The experiment was run on two days, 48 h apart. On day 1, all the experimental groups received the List 1 training and were tested on day 2 (first for List 1 and after that for List 2). The groups differed in the moment they received the interference task (5 min, 30 min, 3 h or 8 h after List 1 training). The “CTL-L1” and “CTL-L2” groups were only trained and tested in one list (List 1 and List 2, respectively). (B) Number of correct responses in the last List 2 training trial on day 1 ± SEM and number of correct responses in the first List 2 testing trial on day 3 ± SEM. (C) List 2 memory change. Mean memory change (number of correct responses at the first List 2 testing trial minus the number of correct responses at the last List 2 training trial) ± SEM. Inset: List 2 mean memory change was fitted to a cubic function: Time is displayed on a logarithmic scale. (D) Number of correct responses in the last List 1 training trial on day 1 ± SEM and number of correct responses in the first List 1 testing trial on day 3 ± SEM (E) List 1 memory change. Memory change mean ± SEM. Inset: List 1 mean memory change was fitted to a cubic function: Time is displayed on a logarithmic scale. * p < 0.05; **, p < 0.01; ***, p < 0.001.

A repeated measures ANOVA analysis revealed “group” per “session” interaction (F _group*session_ (4,110) = 5.40, p = 0.001, η^2^_p_ = 0.16). Thus, we performed simple effects analyses of “group” within each level of “session” followed by pairwise comparisons corrected by Bonferroni’s adjustment for multiple comparisons. First, we showed that all groups (Fig 3A) had the same performance in the last List 2 training trial on day 1 (Fig 3B, “CTL-L2”: 4.30 ± 0.18, “G-5min”: 4.52 ± 0.14. “G-30min”: 4.70 ± 0.10, “G-3h”: 4.57 ± 0.15, “G-8h”: 4.48 ± 0.16, simple effects: F (4,110) = 0.90, p = 0.47). However, they significantly differ in the first List 2 testing trial on day 3 (“CTL-L2”: 2.96 ± 0.24, “G-5min”: 2.39 ± 0.27. “G-30min”: 1.89 ± 0.30, “G-3h”: 2.13 ± 0.25, “G-8h”: 1.52 ± 0.24, simple effects: F (4,110) = 4.30 p = 0.003, η^2^_p_ = 0.13). Specifically, the “G-5min” and “G-3h” groups did not significantly differ from the control “CTL-L2” group that was only trained and tested on List 2 (p = 1.00 and p = 0.27, respectively), evidencing no RIF effect. However, the “G-30min” as well as the “G-8h” groups showed a significantly worse performance than the “CTL-L2” group, evidencing the RIF effect (p = 0.04, d = 0.85 and p = 0.002, d = 1.26, respectively).

Simple effects of session within each level of group showed that there was a significant memory decay between the last List 2 training trial and the first List 2 testing trial for all groups (simple effects, “CTL-L2”: F(1,110) = 23.72, p< 0.001, η^2^_p_ = 0.17; “G-5min”: F(1,110) = 59.27, p < 0.001, η^2^_p_ = 0.35; “G-30min”: F(1,110) = 104.30, p < 0.001, η^2^_p_ = 0.49; “G-3h”: F(1,110) = 77.41, p < 0.001, η^2^_p_ = 0.41; “G-8h”: F (1,110) = 114.14, p < 0.001, η^2^_p_ = 0.51).

We further analyzed the List 2 memory change (number of correct responses at the first testing trial minus the number of correct responses at the last training trial) showing significant differences between groups (Fig 3C, “CTL-L2”: -1.35 ± 0.27, “G-5min”: -2.13 ± 0.30, “G-30min”: -2.83 ± 0.29, “G-3h”: -2.43 ± 0.26, “G-8h”: -2.96 ± 0.26, one-way ANOVA, F (4,114) = 5.39, p = 0.001, η^2^_p_ = 0.16). The “G-5min” and “G-3h” groups showed a similar memory decay than the “CTL-L2” group (Bonferroni, p = 0.48 and p = 0.06, respectively), evidencing no RIF effect, whereas the “G-30min” and the “G-8h” groups showed significantly higher memory decay than the “CTL-L2” group, evidencing RIF effect (p = 0.003, d = 1.12 and p = 0.001, d = 1.30 respectively).

Thus, in both analyses, we observed that the interference task (List 2) presented 5 min or 3 h after List 1 training interfered with the stabilization of the List 1 memory (evidenced by no RIF effect on List 2). However, when the interference task was presented 30 min or 8 h later, it did not impair List 1 memory stabilization (evidenced by RIF effect).

The analysis of the dynamics of List 2 memory change revealed that the curve presented two minimums (higher List 2 memory decay), one at 30 min and the other at 8 h, evidencing higher RIF effect (Fig 3A2 inset).

### List 1 performance

A repeated measures ANOVA revealed “group” per “session” interaction (F_group*session_ (4,110) = 9.45, p < 0.001, η^2^_p_ = 0.25). Thus, we performed simple effects analysis of “group” within each level of “session”. We found no significant differences between groups in the last List 1 training trial on day 1 (Fig 3B1“CTL-L1”: 4.22 ± 0.20, “G-5min”: 4.39 ± 0.17, “G-30min”: 4.26 ± 0.21, “G-3h”: 4.52 ± 0.12, “G-8h”: 4.26 ± 0.20, simple effects F (4,110) = 0.46, p = 0.76). However, there was a simple effect of group within the first List 1 testing session (Fig 3D, CTL-L1”: 2.61 ± 0.29, “G-5min”: 1.22 ± 0.23, “G-30min”: 1.70 ± 0.28, “G-3h”: 0.91 ± 0.17, “G-8h”: 0.83 ± 0.24, F (4,110) = 9.04, p < 0.001, η^2^_p_ = 0.25). Specifically, the “G-5min”, “G-3h” and “G-8h” groups showed significantly worse performance than “CTL-L1” group (p = 0.001, d = 1.14; p < 0.001, d = 1.54 and p < 0.001, d = 1.43, respectively), whereas the “G-30min” group showed a similar performance than the “CTL-L1” group (p = 0.091).

Simple effects of session within each level of group showed that there was a significant memory decay between the last List 1 training trial and the first List 1 testing trial for all groups (Simple effects, “CTL-L1”: F(1,110) = 36.87, p < 0.001, η^2^_p_ = 0.25; “G-5min”: F(1,110) = 143.53, p < 0.001, η^2^_p_ = 0.56; “G-30min”: F (1,110) = 93.76, p < 0.001, η^2^_p_ = 0.46; “G-3h”: F(1,110) = 185.55, p < 0.001, η^2^_p_ = 0.63; “G-8h”: F (1,110) = 168.10, p < 0.001, η^2^_p_ = 0.60).

Furthermore, there were significant differences between groups at List 1 memory change (Fig 3D, “CTL-L1”: -1.61 ± 0.26, “G-5min”: -3.17 ± 0.21, “G-30min”: -2.57 ± 0.29, “G-3h”: -3.57 ± 0.23, “G-8h”: -3.43 ± 0.32, one way ANOVA, F(4,114) = 9.17, p < 0.001, η^2^_p_ = 0.25). Specifically, the “G-5min”, “G-3h” and “G-8h” groups showed a significant higher decay than the “CTL-L1” group (Bonferroni, p = 0.001, d = 1.44, p < 0.001, d = 1.70 and p < 0.001, d = 1.35 respectively). The “G-30min” and the “CTL-L1” groups did not significantly differ (Bonferroni p = 0.12).

Furthermore, an analysis of the dynamics of List 1 memory change showed that the curve presented a maximum at 30 min, evidencing a time point where the memory would be more protected against simultaneous interferences (Fig 3B2 inset).

### Type of errors

We further analyzed the different memory change for each type of error for each list. Regarding the List 2 “void” type errors (when participants did not write any response, or they wrote one letter or two), there was a significant difference between groups (S1A Fig, “CTL-L2”: 0.57 ± 0.21, “G-5min”: 1.43 ± 0.27, “G-30min”: 2.22 ± 0.31, “G-3h”: 1.17 ± 0.26, “G-8h”: 1.70 ± 0.30, F (4,114) = 5.02, p = 0.001, η^2^_p_ = 0.15). We observed that the “G-30min” and “G-8h” groups showed a significantly higher increase in “void” type errors from the last training trial to the first testing trial than the “CTL-L2’’ (Bonferroni, p < 0.001, d = 1.34 and p = 0.04, d = 0.93, respectively). However, there were no significant differences for the “G-5min” and “G-3h” compared to the “CTL-L2” group (p = 0.26 and p = 1, respectively). There were no significant differences between groups neither for the confusion (writing a response-syllable that did not belong to any of the lists, F(4,114) = 1.45, p = 0.22), and intralist (writing a response-syllable that was previously associated to another cue-syllable of the same list, χ*2*(4) = 4.66, p = 0.32).

For List 1, there was a significant difference between groups for the “void” type errors (S1B Fig, “CTL-L1”: 0.74 ± 0.21, “G-5min”: 1.70 ± 0.28, “G-30min”: 2.04 ± 0.29, “G-3h”: 1.65 ± 0.32, “G-8h”: 2.16 ± 0.35, one way ANOVA, F(4,114) = 3.49, p = 0.01, η^2^_p_ = 0.11). Specifically, the “G-30min” and “G-8h” groups showed higher increase of “void” type errors that “CTL-L1” group (Bonferroni, p = 0.023, d = 1.09 and p = 0.012, d = 1.02, respectively). However, no significant differences were found for the “G-5min” and “G-3h” groups compared to the “CTL-L1” (Bonferroni, p = 0.24 and p = 0.31, respectively). There were no significant differences between groups neither for the confusion nor for the intralist type of errors (F(4,114) = 1.73, p = 0.15; F(4,114) = 0.83, p = 0.51, respectively).

### Emotional variables and questionnaires

There were no significant differences between groups at STAI State Anxiety (F(5,137) = 0.30, p = 0.92), STAI Trait Anxiety (F(5,137) = 1.27, p = 0.28), BDI-II (F(5,137) = 0.74, p = 0.60), PSQI (F(5,137) = 0.99, p = 0.43), and MEQ (F(5,137) = 0.68, p = 0.64) (Table 1).

**Table 1:** Emotional variables and questionnaires.

	Stai Anxiety	Stai Trait	BDI-II	PSQI	MEQ
**CTL-L1**	38.35 ± 1.95	43.13 ± 2.58	14.39 ± 2.13	7.65 ± 0.75	43.43 ± 2.20
**CTL-L2**	38.26 ± 1.63	45.09 ± 1.95	13.43 ± 2.12	6.22 ± 0.62	44.22 ± 1.97
**G-5min**	38.22 ± 1.66	41.57 ± 1.97	12.96 ± 1.37	6.61 ± 0.69	45.83 ± 2.21
**G-30min**	37.26 ± 1.17	39.22 ± 1.83	11.09 ± 1.39	7.22 ± 0.74	47.43 ± 1.59
**G-3h**	36.26 ± 1.47	39.17 ± 2.24	11.70 ± 1.27	5.83 ± 0.52	42.74 ± 2.20
**G-8h**	36.87± 1.69	42.61 ± 1.	10.83 ± 1.36	6.96 ± 0.69	44.78 ± 2.11

Mean state anxiety, trait anxiety, depression, sleep quality and chronotype ± SEM.

## Discussion

In the present study, we showed that the dynamics of declarative memory consolidation is not a linear process. As in previous studies, we found that 5 min after learning, as well as 3 h after, the memory was labile and could be interfered by a second learning task that acted as an amnesic agent [4,16,18]. However, we have not only replicated previous results showing that after longer periods (> 6 h) the declarative memory was already stabilized [4] but we also found a new time window, shortly after acquisition (i.e. 30 min), where the memory became rapidly protected against interference. That is, we demonstrated that there are at least two time windows of susceptibility to interference after learning without the external induction of memory reactivation.

To evaluate the existence of memory consolidation/reconsolidation, amnesic agents such as electroconvulsive shock (ECS, [34–37]), protein synthesis and gene expression inhibitors [38,39] and β-blockers [40–42] have been administered after learning/reactivation. However, a more ecological way is to use other learnings [4,5,7,8,43–47]. The use of a training session as an amnesic agent has the advantage of being free of undesirable metabolic side effects or physical perturbations [4]. Moreover, it shares several parameters of the experimental context as well as the pattern of syllable presentation, thus involving related memories. This gave us the possibility of disclosing retrieval interferences during testing and allowed the use of the Retrieval-Induced Forgetting effect (RIF) to demonstrate List 1 memory impairment [4].

We observed List 2 RIF effect on day 3 for the groups that received the List 2 training 30 min as well as 8 h after List 1 training, indicating successful stabilization of List 1 memory trace in both conditions. On the contrary, the List 2 RIF effect was absent on day 3 for the groups that received the List 2 training 5 min or 3 h after List 1 training, suggesting that List 1 memory trace was not yet stabilized at those time points and thus, it was sensitive to disruption by interference learning (Fig 3A1). Furthermore, the same profile of results was obtained by analyzing the List 2 memory change. That is, higher memory decay was observed for the groups that received the List 2 training 30 min as well as 8 h after List 1 training, indicating List 2 RIF effect whereas the groups that received the List 2 training 5 min or 3 h after List 1 training showed a similar memory decay that the control group, evidencing the List 2 RIF effect was absent. Moreover, these results are supported by the interpolation analysis (Fig 3A2 *inset*), which evidences two maximum peaks representing the “G-5min” and “G-3h” groups (susceptible to interference) and two minimum peaks representing the “G-30min” and “G-8h” groups (more protected against interference).

In addition to the RIF effect, we also evaluated List 1 memory performance. Considering our previous results showing that the List 1 memory performance at testing of the groups that received an interference task had significantly more errors than the control group that was only trained and tested in one list. This fact could be explained by simultaneous retrieval interferences of List 1 and List 2 memory at testing or List 1 consolidation/reconsolidation impairment [4,16,18]. Thus, here we expected a reduction in List 1 memory performance at testing for all groups compared to the control condition. Nevertheless, contrary to our expectations, the group that received the interference task 30 min after List 1 training showed a similar performance to the “CTL-L1” group, evidencing differential outcomes for this temporal window not only through the List 2 RIF effect but also in List 1 memory retrieval. Furthermore, the analysis of List 1 memory change showed the same results. That is, the group that received the List 2 after 30 min of List 1 training presented similar memory decay that the “CTL-L1” group whereas the other groups showed higher memory decay than the control one. Moreover, these results are supported by the interpolation analysis (Fig 3B2 *inset*), which shows one maximum peak at 30 min after acquisition, indicating that the memory is more protected against interference. Taken together, these results challenge previous hypotheses establishing that consolidation is a linear process that begins after learning and that once it is accomplished, memories are protected against interference [4,5,48]. However, our findings differ from those of Kaczer et al. [7] who found that consolidation of new words was interfered only immediately after learning but not 30 min, 4 h or 24 h later. This could be due to differences in the learned material. It has been proposed that new words could be acquired without relying on the hippocampus and rapidly integrated into the neocortex in a process known as ‘fast mapping’ [49].

In addition, when we evaluated the memory change regarding the types of errors, we found the same pattern of results for “Void” type errors in both lists. The “G-30min” and the “G-8h” groups showed a significantly higher increase in this type of error compared to the correspondent control group. We have previously discussed that the “Void” type error, that is not writing any syllable or just one or two letters of the triplet could be thought of as an effect of the natural forgetting [18] or also an effect of forgetting caused by retrieval interferences such as simultaneous retrieval of related information or retrieval-induced forgetting (tip of the tongue" effect, i.e. trying to access stored information but not being able to do so. That is, not being able to respond when the cue syllable appears).

At a molecular level, it is interesting to note that this short time window after acquisition, where the declarative memory seems to be transiently protected against interferences, matches the early consolidation processes that take place within about 30 minutes and induce a fast increase in synaptic strength independent of protein synthesis [50,51]. However, these early changes are transient and decay after about 90 minutes [52]. So, it is possible to speculate that within about 30 minutes after List 1 acquisition, a rapid stabilization, independent of protein synthesis, occurs protecting the memory against List 2 interference. Nevertheless, this does not explain the results underlying the absence of simultaneous retrieval interferences on List 1 for the “G-30min” group, that is, the no significant difference between “G-30min” and “CTL-L1” group as we have previously observed for other time windows [4]. Therefore, we suggest that not only synaptic consolidation would be involved, but also that a rapid system consolidation process could be initiated during learning or shortly after acquisition has ended [53,54]. Brodt et al. [54] using an object–location association task, observed neocortical plasticity (specifically in the parietal cortex) as early as 1 h after learning and found that it was learning specific. They suggested that new traces are encoded rapidly in the neocortex from the learning onset, challenging traditional models of slow systems consolidation. In the same line, studies in rodents have revealed that neocortical cells are already tagged during encoding and have detected experience-dependent microstructural changes as early as 1 h after learning [55–57]. However, these studies do not explain the possibility of multiple time windows of susceptibility after acquisition. In this line, Dudai and Eisenberg [12] and Alberini [3] has previously described a model that postulates that memory reconsolidation—i.e. the restabilization of the memory trace that follows the labilization induced by its non-reinforced retrieval—could be just a manifestation of a lingering consolidation process. According to this model, consolidation may include several subsequent reactivation events whose function is to further strengthen and/or prolong memory retention. This hypothesis predicts that there should be recurrent time windows of susceptibility to consolidation blockers over hours, days, or weeks. Taking all these into account, we speculate that this transient stabilization period (30 min) could be the consequence of the interplay between rapid system consolidation processes initiated during learning and synaptic consolidation in hippocampal and neocortical stores. That is, List 1 memory could be rapidly integrated at the neocortical level, perhaps involving the induction of a fast increase in synaptic strength independent of protein synthesis (around 30 min after learning), protecting it against interference. Followed by this early consolidation process, the strength of the trace could decay, becoming again dependent on protein synthesis and susceptible to interferences.

The lack of measures of the participant´s level of alertness and attention leave out the possibility to discriminate the impact of these variables, adding further limitations to the study. Finally, although we used a scale to measure sleep quality (PSQI), no objective measures of sleep quality were used, adding further limitations to the study.

Taking the present results into account, we propose that the declarative memories are not protected against interference after a unique time window during the first 24 h after training, on the contrary, we found that there are at least two time windows of susceptibility. Shen et al. [8] showed that after memory reactivation a second task could interfere with memory re-stabilization up to 20 min, but no interference was observed after 30 and 40 min proposing that the memory was protected against interference 30 min after acquisition. However, they did not study the effect of interference after longer periods. Thus, considering that consolidation and reconsolidation share similar molecular mechanisms it would be of great interest for the clinical field to study this short time window where the memory is protected against interference. In the last few years, lots of studies have proposed the possibility of using reconsolidation as a therapeutic tool for the intervention of maladaptive memories [42,58–64]. That is, to reactivate a maladaptive memory and update its content. Our results suggest that the time window where the therapeutic intervention is administered should be carefully taken into account because the time windows of susceptibility seem not to be a linear process, at least regarding memory consolidation. Thus, due to the importance of this short time window of stabilization/restabilization for the clinical field, we suggest that more studies should be done using direct methods for the consolidation/reconsolidation of declarative memories. The use of an indirect method (RIF) is one limitation of the present study. Further experiments should be conducted to understand the consolidation and reconsolidation dynamics.

## Supporting information

S1 Fig Memory change of “void” type errors. (A) Mean memory change of List 2 void type errors (number of void type errors at the first List 2 testing trial minus the number of void type errors at the last List 2 training trial) ± SEM. (B) Mean memory change of List 1 void type errors ± SEM.
(TIF)
