# Supplementary figures and images for: Non-linear susceptibility to interferences in declarative memory formation
Source: PLoS One. 2022 Jun 29;17(6):e0270678. doi: 10.1371/journal.pone.0270678 (PMC9242448; doi:10.1371/journal.pone.0270678)

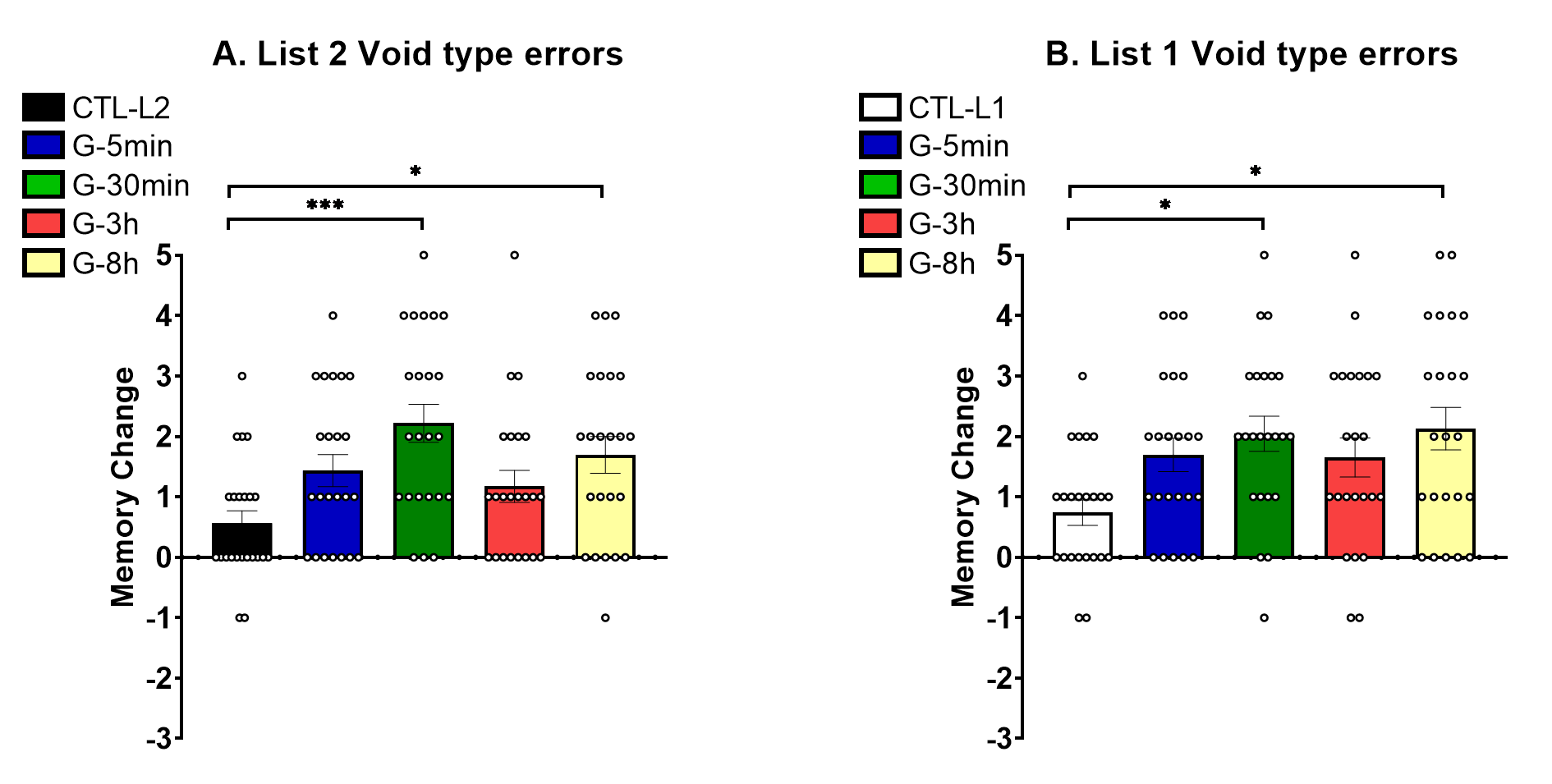

Supplement: S1 Fig — (A) Mean memory change of List 2 void type errors (number of void type errors at the first List 2 testing trial minus the number of void type errors at the last List 2 training trial) ± SEM. (B) Mean memory change of List 1 void type errors ± SEM. (TIF) [file pone.0270678.s001.tif]
